# Oleacein Attenuates Lipopolysaccharide-Induced Inflammation in THP-1-Derived Macrophages by the Inhibition of TLR4/MyD88/NF-κB Pathway †

**DOI:** 10.3390/ijms23031206

**Published:** 2022-01-21

**Authors:** Santa Cirmi, Alessandro Maugeri, Caterina Russo, Laura Musumeci, Michele Navarra, Giovanni Enrico Lombardo

**Affiliations:** 1 Department of Chemical, Biological, Pharmaceutical and Environmental Sciences, University of Messina, 98166 Messina, Italy; scirmi@unime.it (S.C.); amaugeri@unime.it (A.M.); carusso@unime.it (C.R.); laura.musumeci@unime.it (L.M.); gelombardo@unime.it (G.E.L.); 2 Department of Pharmacy-Drug Sciences, University of Bari “Aldo Moro”, 70125 Bari, Italy; 3 Fondazione “Prof. Antonio Imbesi”, 98168 Messina, Italy

**Keywords:** oleacein, *Olea europea*, inflammation, LPS, THP-1 monocytes, CD14, TLR4, NF-κB, MyD88, ROS

## Abstract

It is known that plant phenolic compounds exert anti-inflammatory activity through both anti-oxidant effects and modulation of pivotal pro-inflammatory factors. Recently, *Olea europaea* has been studied as a natural source of bioactive molecules; however, few studies have focused on the biological effect of oleacein (OLC), the most abundant secoiridoid. Therefore, the aim of this study was to investigate the potential anti-oxidant activity of OLC, as well as to study its anti-inflammatory effect in lipopolysaccharide (LPS)-stimulated THP-1-derived macrophages. LPS brought a dramatic increase of both release and gene expression of pro-inflammatory cytokines (IL-6, IL-1β and TNF-α), as well as a decrease of anti-inflammatory ones (IL-10), the effects of which are reverted by OLC. Moreover, it reduced the levels of COX-2, NO and PGE_2_ elicited by LPS exposure in THP-1 macrophages. Interestingly, OLC modulated inflammatory signaling pathways through the inhibition of CD14/TLR4/CD14/MyD88 axis and the activation of NF-κB. Finally, OLC showed relevant anti-oxidant capability, assessed by abiotic assays, and reduced the intracellular amount of ROS generated by LPS exposure in THP-1 macrophages. Overall, these results suggest that the anti-oxidant activity and anti-inflammatory effect of OLC may cooperate in its protective effect against inflammatory stressors, thus being a possible alternative pharmacological strategy aimed at reducing the inflammatory process.

## 1. Introduction

Inflammation is an adaptive process of the immune system, prompted by harmful stimuli, which starts in order to initiate the healing process [1,2]. It is well-recognized that inflammation is closely related to tumor proliferation and migration [3], as well as in many other diseases, such as neurodegenerative [4], cardiovascular [5], diabetic [6] and bowel diseases [7,8].

Macrophages play a pivotal role in the initiation, maintenance and resolution of inflammation through the production of different cytokines and growth factors. They can be activated in M1 macrophages when stimulated by IFN-γ or lipopolysaccharide (LPS), or M2 macrophage if they are activated by IL-4 and IL-13 [9]. The activated M1 macrophages promote inflammation by producing and releasing cytokines, such as interleukin-1 beta (IL-1β), interleukin-6 (IL-6) and tumor necrosis factor-alpha (TNF-α), along with nitric oxide (NO) and prostaglandins (PGE2) [10]. The latter are mediators of inflammatory response synthesized starting from arachidonic acid by both isoforms of cyclooxygenase (COX), COX-1 and COX-2. Therefore, these can be considered the main targets of anti-inflammatory therapy, which are successfully inhibited by nonsteroidal anti-inflammatory drugs (NSAIDs). However, along with NSAIDs, several classes of drugs are used to manage the inflammatory diseases, such as corticosteroids and biologics. Nevertheless, these drugs are not free from side effects. To restrict these disadvantages, in the last decades, the use of natural remedies, nutraceuticals and food supplements has increased significantly as an alternative and/or complementary medicine to treat inflammation [11,12]. This is because, even if natural products are not risk-free, commonly, they are safer than both synthetic and biologic drugs.

Polyphenols are plant secondary metabolites, which, in the last decades, attracted the attention of the scientific community due to their health properties. Several epidemiological studies and meta-analyses strongly suggest that long-term consumption of diets rich in plant polyphenols offer protection against development of cancers, cardiovascular diseases, diabetes, osteoporosis and neurodegenerative diseases [13,14,15,16,17]. A major source of polyphenols (40–1000 mg/kg) is represented by extra virgin olive oil (EVOO), whose phenolic fraction includes simple phenols (tyrosol and hydroxytyrosol), secoiridoids (oleuropein, oleocanthal and oleacein) and lignans [18].

Oleacein is the dialdehydic form of the decarboxymethyl elenolic acid esterified with 3,4-(dihydroxyphenyl) ethanol (3,4-DHPEA-EDA; OLC), which is the predominant compound of fruit and leaves of *Olea europaea* L. In the past decades, OLC attracted interest of scientific community due to its proved bioactivity. It showed protective effects against abdominal fat accumulation, weight gain and liver steatosis with improvements in insulin-dependent glucose and lipid [19,20]. It was also found that OLC protects endothelial progenitor cells (EPCs) against pathogenic effects of angiotensin II by increase of heme oxygenase 1 expression and restore the EPC ability of neovascularization and angiogenesis [21]. In addition, OLC mitigates the destabilization of carotid plaque [22], exerts anti-microbial effects [23] and possesses anti-estrogenic effect [24]. Moreover, the activity of OLC against cancer has been widely proven in several in vitro models, among which neuroblastoma [25], leukemia [26], multiple myeloma [27] and melanoma [28] or non-melanoma skin [29] cancer. 

Several research groups reported the anti-oxidant and anti-inflammatory activities of oleocanthal and oleuropein [30,31,32,33,34,35,36,37], while those of OLC are still poorly defined. Recently, it has been shown that OLC exerts anti-inflammatory effects in human adipocytes challenged with TNF-α, a prototypic inflammatory stimulus, which could aid to explain the cardiometabolic benefit of EVOO consumption [32]. However, to date, there are no data on the effects of OLC on LPS-induced inflammation. In this study, we evaluated the potential anti-inflammatory effect of OLC in LPS-stimulated THP-1-derived macrophages, which represent a well-established model to study inflammation in vitro and the involved molecular mechanisms.

## 2. Results

### 2.1. Effect on Cell Viability of THP-1 Cells

**Figure 1:**
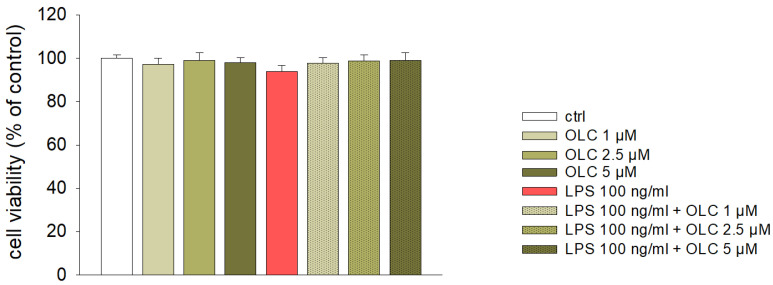
Effect of OLC in THP-1-derived macrophages viability in presence or absence of LPS. Different concentrations of OLC (1, 2.5 and 5 μM) were added to culture medium 30 min before LPS treatment (100 ng/mL for 24 h), and then cell viability was assessed by the MTT test. The results are expressed as the percentage of viable cells in treated cultures compared to the percentage in untreated ones. Data are means ± SEM from three independent experiments performed in eight replicates.

To exclude any cytotoxic effect of OLC on THP-1-derived macrophages and to establish its non-lethal concentrations, preliminary experiments were performed using the 1-(4,5-dimethylthiazol-2-yl)-3,5-diphenylformazan (MTT) test. The results of this assay shown that pre-treatment of THP-1-derived macrophages with different concentrations of OLC (1–50 µM) for 30 min prior the exposure to LPS for 24 h did not hamper cell viability up to 5 µM (Figure 1) in presence or absence of LPS. However, cell viability significantly decreased from 10 to 50 μM (data not shown). These results indicate that OLC is non-toxic up to 5 µM, and hence, these concentrations were used for further experiments.

### 2.2. Effect of OLC on the Production of Inflammatory Cytokines in LPS-Stimulated THP-1 Cells

**Figure 2:**
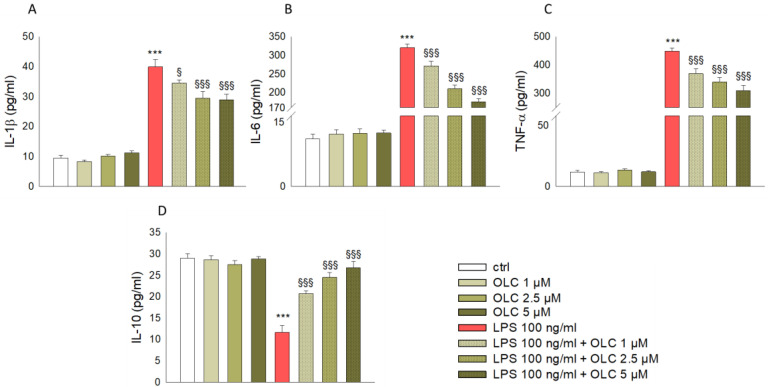
OLC reduced the release of IL-1β, IL-6 and TNF-α and enhanced that of IL-10 in LPS-stimulated THP-1-derived macrophages. The cells were treated with increasing concentrations of OLC (1, 2.5 and 5 μM) for 30 min prior LPS (100 ng/mL) for 24 h. Then, secretion of IL-1β (**A**), IL-6 (**B**), TNF-α (**C**) and IL-10 (**D**) in the cultured medium was evaluated by ELISA assay. Data are the mean ± SEM of three independent experiments performed in triplicate. *** *p* < 0.001 vs. ctrl; ^§^ *p* < 0.05 vs. LPS-treated cells; ^§§§^ *p* < 0.001 vs. LPS-treated cells.

To understand the effect of OLC on LPS-stimulated IL-1β, IL-6, TNF-α and IL-10 production, the supernatants from the THP-1-derived macrophages were collected at the end of the treatment and were assayed using ELISA kits for the respective cytokines. As shown in Figure 2, treatment of THP-1-derived macrophages with LPS alone for 24 h resulted in augmented release of IL-1β (*p* < 0.001; Figure 2A), IL-6 (*p* < 0.001; Figure 2B) and TNF-α (*p* < 0.001; Figure 2C) accompanied by a reduced release of IL-10 (*p* < 0.001; Figure 2D) compared to control cells. Pre-treatment with OLC decreased the overproduction of IL-1β (*p* < 0.05 for OLC 1 μM and *p* < 0.001 for OLC 2.5 and 5 μM; Figure 2A), IL-6 (*p* < 0.001; Figure 2B) and TNF-α (*p* < 0.001; Figure 2C) pro-inflammatory cytokines in LPS-activated THP-1-derived macrophages. On the contrary, the production of the anti-inflammatory cytokine IL-10 was significantly enhanced after THP-1 exposure to OLC (*p* < 0.001; Figure 2D). 

### 2.3. Effect of OLC on LPS-Induced Inflammatory Gene Transcription

**Figure 3:**
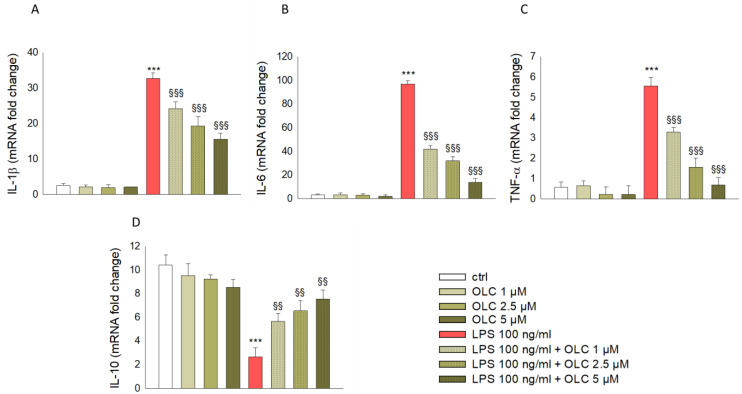
Effects of OLC on cytokine gene expression in THP-1-derived macrophages stimulated with LPS. THP-1 cells were treated with different concentrations of OLC (1, 2.5 and 5 μM) for 30 min prior LPS (100 ng/mL) for 24 h. The results from real-time PCR of IL-1β (**A**), IL-6 (**B**), TNF- α (**C**) and IL-10 (**D**) are expressed as a relative fold change compared to untreated cells, after normalization against β-actin as endogenous control. Data are the mean ± SEM of three independent experiments performed in triplicate. *** *p* < 0.001 vs. ctrl; ^§§^ *p* < 0.01 vs. LPS-treated cells; ^§§§^ *p* < 0.001 vs. LPS-treated cells.

In order to confirm the LPS-induced release of both pro- and anti-inflammatory cytokines, we evaluated the gene levels of the above-mentioned cytokines by real-time PCR. As shown in Figure 3, LPS stimulation of THP-1-derived macrophages resulted in a huge increase of the mRNA levels of IL-1β (*p* < 0.001; Figure 3A), IL-6 (*p* < 0.001; Figure 3B) and TNF-α (*p* < 0.001; Figure 3C), along with a decrease of those of IL-10 (*p* < 0.001; Figure 3D). Pretreatment of THP-1-derived macrophages with OLC strongly suppressed the mRNA expression of these inflammatory mediators in a concentration-dependent manner, with the highest effect at 5 μM (Figure 3). In particular, the IL-6 mRNA increase was higher than that observed for IL-1β and TNF-α; it was almost 95-fold greater than that found in control cells (Figure 3B). This effect was counteracted by pre-treatment with OLC at all the tested concentration (*p* < 0.001; Figure 3B). In parallel, the LPS-induced increase in IL-1β (~30-fold higher than controls) and TNF-α (~5-fold higher than controls) gene expression was significantly reduced by each OLC concentration used in this study (*p* < 0.001; Figure 3A,C). On the contrary, the expression of anti-inflammatory IL-10 gene was significantly enhanced after THP-1-derived macrophages were exposed to all concentrations of OLC (*p* < 0.01; Figure 3D). 

### 2.4. Inhibitory Effect of OLC on NO, COX-2 and PGE2 Production in LPS-Stimulated THP-1 Cells

**Figure 4:**
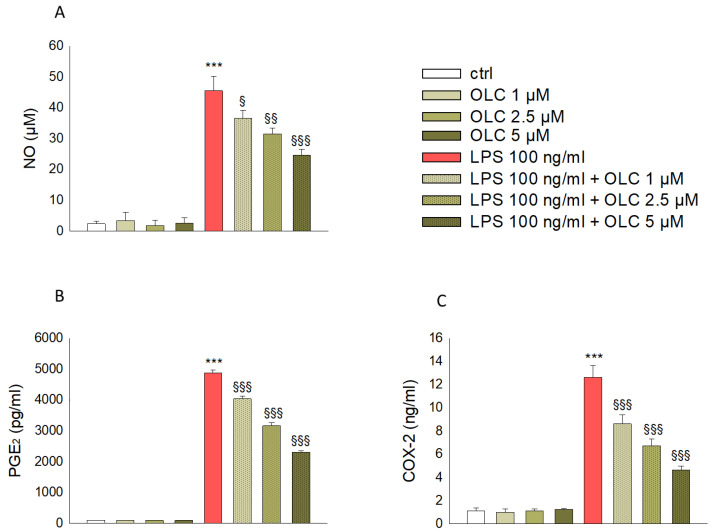
Effects of OLC in LPS-induced production of pro-inflammatory mediators in THP-1-derived macrophages. THP-1-derived macrophages were treated with different concentrations of OLC (1, 2.5 and 5 μM) for 30 min prior LPS (100 ng/mL) for 24 h. The results from the Griess reaction of NO (**A**) and ELISA assay of PGE_2_ (**B**) and COX-2 (**C**) are shown. Data are the mean ± SEM of three independent experiments performed in triplicate. *** *p* < 0.001 vs. ctrl; ^§^ *p* < 0.05 vs. LPS-treated cells; ^§§^ *p* < 0.01 vs. LPS-treated cells; ^§§§^ *p* < 0.001 vs. LPS-treated cells.

To verify the potential anti-inflammatory activity of OLC on THP-1-derived macrophages, the levels of the mediators NO, COX-2 and PGE2, secreted after LPS stimulation, were determined. As showed in Figure 4A, LPS stimulation of THP-1-derived macrophages caused a significant increase in NO release (*p* < 0.001), as evaluated by a Griess reaction. This effect was significantly counteracted by pre-treatment with OLC at all tested concentrations (1, 2.5 and 5 μM) in a concentration-dependent manner (*p* < 0.05 for OLC 1 μM; *p* < 0.01 for 2.5 μM and *p* < 0.001 for 5 μM). The inhibitory effect of OLC on COX-2 and PGE_2_ production in LPS-activated THP-1-derived macrophages was determined by ELISA. Figure 4B,C showed, respectively, that OLC inhibited PGE2 (*p* < 0.001) and COX-2 (*p* < 0.001) production in LPS-stimulated THP-1-derived macrophages in a concentration-dependent manner. These results indicate that OLC effectively counteracted the production of NO and COX-2, which might cause the reduced synthesis of PGE_2_. 

### 2.5. OLC Down-Regulated Cellular Expression of Both CD14 and TLR4 in LPS-Stressed THP-1-Derived Macrophages

**Figure 5:**
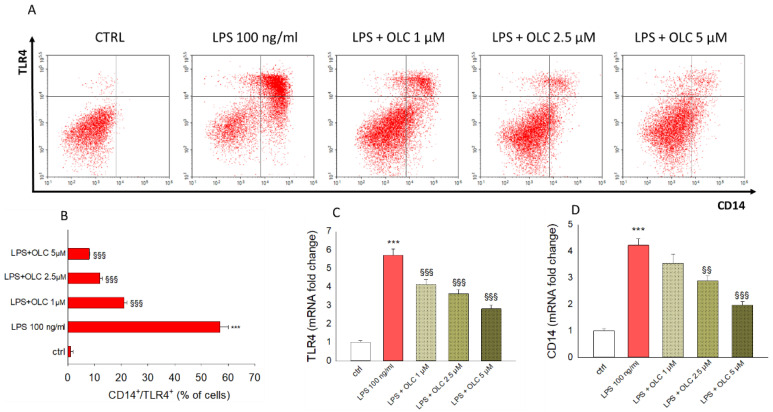
Effects of OLC on the expression of TLR4 and CD14 in THP-1 differentiated cells exposed to LPS. The evaluation of the expression of the two membrane markers was performed by a two-staining flow cytometry method (PE-TLR4 and FITC-CD14) and real-time PCR. The dot plots (**A**) are representative of three separate experiments executed in triplicate (*n* = 9). The histograms (**B**) show the percentage ± SEM of cells positive for both TLR4 and CD14 staining. The results from the real-time PCR of TLR4 (**C**) and CD14 (**D**) are expressed as a relative fold change compared to untreated cells, after normalization against β-actin as endogenous control. *** *p* < 0.001 vs. ctrl; ^§§^ *p* < 0.01 vs. LPS-treated cells; ^§§§^ *p* < 0.001 vs. LPS-treated cells.

The significant stimulation of both CD14 and TLR4 expression induced by LPS in differentiated THP-1 cells was clear by the shift of cell population towards the upper-right quadrant of the dot plot respect unstimulated cells (Figure 5A). In detail, the 57 ± 3% of cells populate this quadrant after LPS exposure, an effect counteracted by the treatment with OLC at 1, 2.5 and 5 µM, which brought the percentages of cells in the upper-right quadrant to the 21 ± 1%, 12 ± 0.9% and 8 ± 0.2%, respectively, showing a down-regulation of both CD14 and TLR4 expression (Figure 5A,B). Figure 5C,D reflect that the expression levels of TLR4 (Figure 5C) and CD14 (Figure 5D) receptors in the LPS-treated THP-1-derived macrophages were dramatically increased (*p* < 0.001). This effect was hampered by pre-treatment with OLC at all the tested concentrations (*p* < 0.001 for TLR4; *p* < 0.01 and *p* < 0.001 for OLC 2.5 μM and 5 μM for the expression of CD14, respectively). 

### 2.6. OLC Attenuate LPS-Induced Activation of MyD88 and NF-κB in THP-1-Derived Macrophages

**Figure 6:**
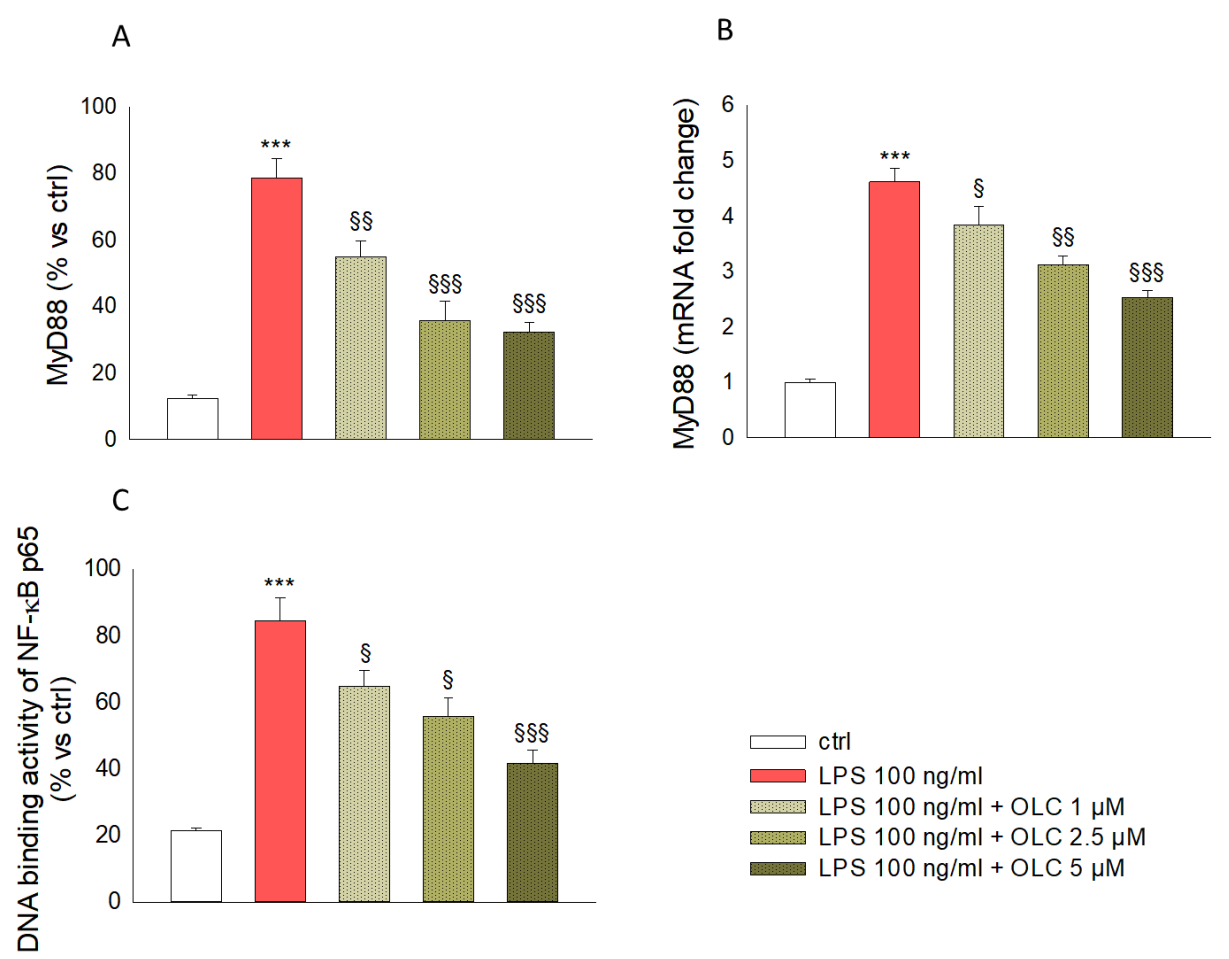
Modulation of LPS-induced activation of MyD88 and NF-κB in THP-1-derived macrophages by OLC. THP-1-derived macrophages were treated with different concentrations of OLC (1, 2.5 and 5 μM) for 30 min prior LPS (100 ng/mL) for 24 h. The results from MyD88 measured by ELISA assay (**A**) and real-time PCR (**B**) are shown along with results of DNA binding activity of NF-κB p65 (**C**) obtained by ELISA tests. Data are the mean ± SEM of three independent experiments performed in triplicate. *** *p* < 0.001 vs. ctrl; ^§^ *p* < 0.05 vs. LPS-treated cells; ^§§^ *p* < 0.01 vs. LPS-treated cells; ^§§§^ *p* < 0.001 vs. LPS-treated cells.

THP-1-derived macrophages were treated as described in the Methods section. As shown in Figure 6A, MyD88 levels were enhanced of about 85% after exposure to LPS compared to control cells (*p* < 0.001). Lower levels of MyD88 were observed in THP-1-derived macrophages pre-treated with OLC before the incubation with LPS. In particular, OLC 1, 2.5 and 5 μM reduced MyD88 levels of about 31% (*p* < 0.01), 56% (*p* < 0.001) and 59% (*p* < 0.001), respectively, compared to LPS-treated cells. A similar trend was also observed at gene level (Figure 6B). In particular, LPS caused a marked increase of MyD88 expression (*p* < 0.001) that was counteracted by pre-treatment with OLC at all tested concentrations (*p* < 0.05 for OLC 1 μM; *p* < 0.05 for OLC 2.5 μM; *p* < 0.001 for OLC 5 μM; Figure 6B).

The ability of the p65 subunit of NF-κB to bind to the DNA consensus site, which may correlate positively with the activation of NF-κB, was quantified in nuclear extracts by an ELISA-based method. As expected, as shown in the Figure 6C, LPS induced a marked increase in the DNA binding activity of the p65 NF-κB subunit (*p* < 0.001). THP-1-derived macrophages exposure to OLC at concentration of 1, 2.5 and 5 μM, reduced NF-κB activation in response to LPS by about 34% (*p* < 0.05), 45% (*p* < 0.05) and 51% (*p* < 0.001), respectively. 

### 2.7. Antioxidant Activity of OLC

The antioxidant and radical scavenging properties of OLC were demonstrated using different assays, as described in the Methods section. As reported in Table 1, the substantial antiradical activity of OLC shown in the 2,2-diphenyl-1-picrylhydrazyl (DPPH) test and expressed as milligrams of Trolox equivalents (TE) per gram of extract (mg TE/g) was 68.37± 1.28, while in the potassium ferricyanide-reducing antioxidant power (PFRAP) test, it was 80.14 ± 1.31, expressed as milligrams of ascorbic acid equivalent (AAE) per gram of OLC (mg AAE/g). Moreover, the obtained values of 70.65 ± 1.13 µmol TE/g OLC demonstrated high antioxidant capacity of OLC against peroxyl radicals (Table 1).

### 2.8. OLC Hampered LPS-Induced ROS Production in THP-1-Derived Macrophages

**Figure 7:**
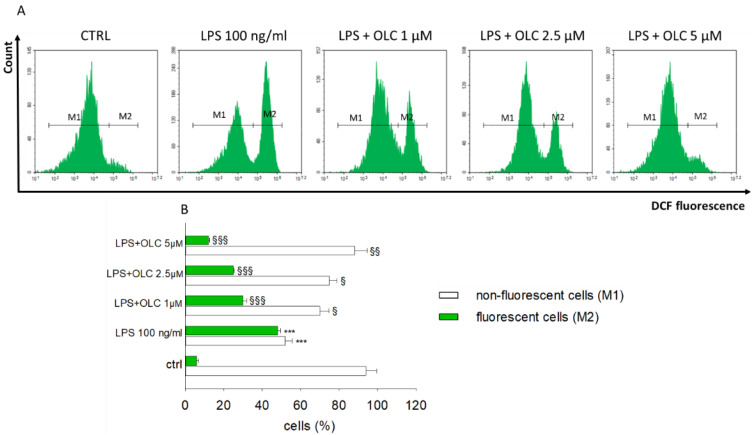
Anti-oxidant effect of OLC in LPS-stressed THP-1-derived macrophages. Levels of intracellular ROS were measured by means of flow cytometry employing DCFH_2_-DA as probe. The plots are representative of three independent experiments (**A**). The histograms (**B**) display the percentage ± SEM of healthy cells (non-fluorescent, M1) and the percentage with increased intracellular ROS (fluorescent, M2) of three separate experiments in triplicate (*n* = 9). *** *p* < 0.001 vs. ctrl; ^§^ *p* < 0.05 vs. LPS-treated cells; ^§§^ *p* < 0.01 vs. LPS-treated cells; ^§§§^ *p* < 0.001 vs. LPS-treated cells.

The exposure to LPS (100 ng/mL) of THP-1-derived macrophages brought a massive increase of intracellular ROS respect to untreated cells, as displayed by the extent of measured fluorescence (Figure 7). In particular, we observed that 48 ± 1.6% of cells showed augmented intracellular ROS after LPS exposure. Contrariwise, the pre-treatment with OLC at 1, 2.5 and 5 µM hampered the oxidant effect of the bacterial endotoxin; thus, the percentage of fluorescent cells were 30 ± 2%, 25 ± 0.5% and 12 ± 0.8%, respectively (Figure 7).

## 3. Discussion

Human THP-1 cells, a human leukemia monocytic cell line, have widely been used as a model to study the immune response capacity of monocytes and monocyte-derived macrophages, because of similarities in their responses when compared to the monocyte fraction present in peripheral blood mononuclear cells (PBMCs) [38]. After treatment with PMA, these cells differentiate into macrophage-like cells. LPS is a type of endotoxin, which can elicit a strong immune response in animals and is widely applied as a pathogenic factor in several research field [39]. Therefore, we investigated the effect of OLC on the inflammatory response in LPS-stimulated THP-1 macrophages.

Firstly, we measured cell viability after OLC treatment in the presence or absence of LPS for 24 h to ascertain its toxicity. OLC demonstrated no cytotoxic effects at concentrations ranging from 1 to 5 μM tested either alone or in the presence of LPS, which were consequently employed in the following experiments.

It is well-known that the pro-inflammatory cytokines are the main contributors of the onset of chronicity and inflammation-associated diseases, through promoting the secretion of other mediators and recruiting macrophages to the site of inflammation, which contribute to the disease worsening [40]. After LPS challenge, the production of pro-inflammatory cytokines IL-1β, IL-6 and TNF-α was markedly increased in THP-1-derived macrophages, along with their gene expression, which was extremely promoted. IL-1β, TNF-α and IL-6 are broadly considered as central markers of inflammatory response and involved in triggering the activation of NF-κB signaling pathway, which in turn promotes the transcription of several factors known to aggravate inflammatory response [41,42]. Hence, it is pivotal to suppress the expression these cytokines. In our study, we observed that OLC suppressed the excessive secretion of IL-1β, TNF-α and IL-6 stimulated by LPS. Simultaneously, we found that the expression level of IL-10 was down-regulated by LPS, while pre-treatment with OLC counteracted this effect. Interestingly, it has been reported that monocytes are primarily responsible for producing IL-10 to block NF-κB activity, thereby suppressing the secretion of proinflammatory cytokines and inhibiting the elicitation of inflammatory cascade [43]. Hence, the increase of IL-10 secretion induced by OLC was conducive to fighting inflammation. A previous study found that OLC significantly decreased expression of pro-inflammatory cytokines (IL-1β and TNF-α), while it increased the anti-inflammatory cytokine IL-10 in an experimental model of autoimmune encephalomyelitis [44].

The overexpression of NO synthase, especially inducible nitric oxide synthase (iNOS), is an important manifestation of LPS-induced stress in macrophages, which leads to the excessive production of NO and, eventually, inflammation [45]. Likewise, COX-2 promotes the release of the pro-inflammatory factor PGE_2,_ sustaining the inflammatory response [46]. In our study, we observed a rise in the levels of NO, COX-2 and PGE_2_ in THP-1-derived macrophages treated with LPS alone. On the contrary, OLC significantly reduced the overexpression of NO, COX-2 and PGE_2_ induced by LPS in THP-1-derived macrophages, thus exhibiting anti-inflammatory effects. In this line, a previous study reported a reduction of NO production by OLC in LPS-stimulated monocytes [47], as well as the reduction of COX-2 and PGE2 increased by LPS in freshly isolated human monocytes [48].

It is well-known that uncontrolled inflammatory cytokine production is closely related to different inflammatory-based diseases (i.e., bronchial asthma, rheumatoid arthritis, psoriasis and sepsis) [40,49] and that their expression is finely modulated by NF-κB [50]. It is a central transcription factor that, generally, migrates within the cytosol bound to IκB, the inhibitor of NF-κB. Following the stimulation, IκB is phosphorylated by IκB kinase and degraded by proteasome. Then, NF-κB is released and moves into the nucleus [51]. Several studies reported that stimulation of THP-1 macrophages with LPS allows the translocation of the p65 subunit of heteromeric NF-κB protein into the nucleus, by phosphorylation and degradation of the IκB subunit, and prompting the expression of various proinflammatory genes by binding to their promoter regions leading to inflammatory response [51]. This is due to the overproduction of inflammatory cytokines, which stimulates the NF-κB signaling and results in the exacerbation of inflammation. In the present study, we observed that LPS induced a marked increase in the DNA binding activity of the p65 NF-κB subunit of THP-1-derived macrophages, an activity that was significantly reversed by OLC treatment. Hence, together with previous results, this suggests that OLC may inhibit the expression of inflammatory cytokines through blocking the NF-κB pathway. 

TLR4 was the first pattern-recognition receptor (PRR) that was identified among TLR family members [52] playing a pivotal role in inflammatory processes [53]. When LPS is released from the bacterial membrane, it is caught by the TLR4-MD-2 heterodimer that has complex ligand specificity, through two accessory proteins: LPS-binding protein and CD14 [54]. Then, the TLR4 dimer binds to MyD88 protein, thereby activating the downstream NF-κB and MAPK signaling pathways [55]. Therefore, the inhibition of this dimer represents an alternative method for treating inflammation-related diseases. In this study, we evaluated the effects of OLC on TLR4 dimerization and the related signaling pathways. We observed a down-regulation of both CD14 and TLR4 expression in THP-1-derived macrophages exposed to OLC prior LPS exposure compared to those exposed to LPS alone. On this basis, we can speculate that the inhibitory effect of OLC on the activation of the NF-κB pathway in THP-1-derived macrophages is ascribable to its inhibitory effect on the expression of TLR4.

**Figure 8:**
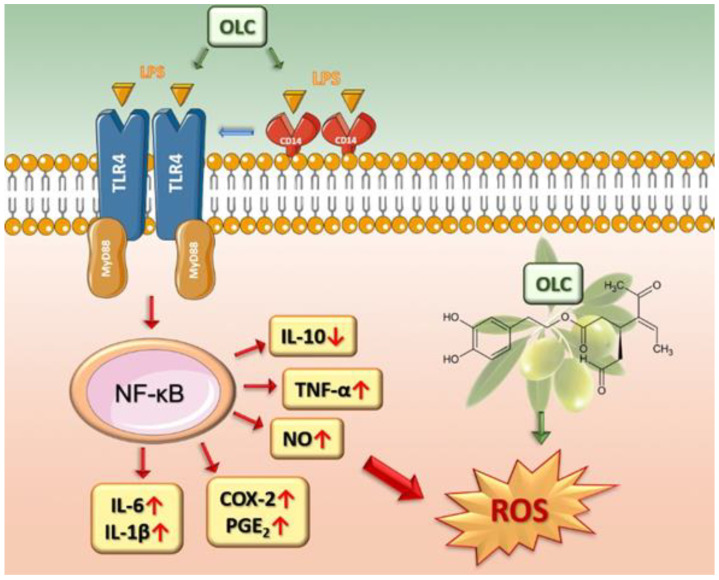
Mechanisms underlying the protective effects of OLC.

Immune cells increase the number of reactive species at the site of inflammation to counteract the noxious stimuli, leading to enormous oxidative stress. Consequently, sustained levels of reactive oxygen/nitrogen species enhance the expression of pro-inflammatory factors, thus starting a vicious cycle [56]. Therefore, inflammation and oxidative stress are strictly linked pathophysiological events, one of which can be easily induced by the other, participating in the pathogenesis of many diseases [57]. In this frame, the lack of drugs that selectively target both oxidative stress and inflammation, the failure to use both antioxidant and anti-inflammatory agents simultaneously or the employment of non-selective agents that block some of the oxidative and/or inflammatory signaling pathways but amplify the others might be responsible for the therapeutic failure in some pathologies. Since activated macrophages release excessive amount of ROS during inflammation, flow cytometric analysis of intracellular ROS levels in LPS-stimulated THP-1 differentiated macrophages demonstrated that pre-treatment with OLC significantly attenuated the LPS-induced excessive ROS generation. Interestingly, OLC proved to also be a great antioxidant in abiotic models, where it was able to quench both oxygen (ORAC) and nitrogen (DPPH) radicals, along with possessing interesting reducing power, as observed by the reduction of ferric ions into ferrous ones. The anti-oxidant and anti-inflammatory effects of OLC are mediated by several mode of action that are gathered in Figure 8.

## 4. Materials and Methods

### 4.1. Cell Culture, Differentiation and Treatments

The human leukemia monocytic cell line THP-1 was originally obtained from ATCC (Rockville, MD, USA). The cells were maintained in RPMI 1640 supplemented with 10% (*v*/*v*) heat-inactivated fetal bovine serum, L-glutamine (2 mM), HEPES (10 mM), sodium pyruvate (1 mM), glucose (2.5 g/L), 2-mercaptoethanol (0.05 mM), penicillin (100 IU/mL) and streptomycin (100 µg/mL) at 37 °C in 5% CO_2_ air humidified atmosphere. 

**Figure 9:**
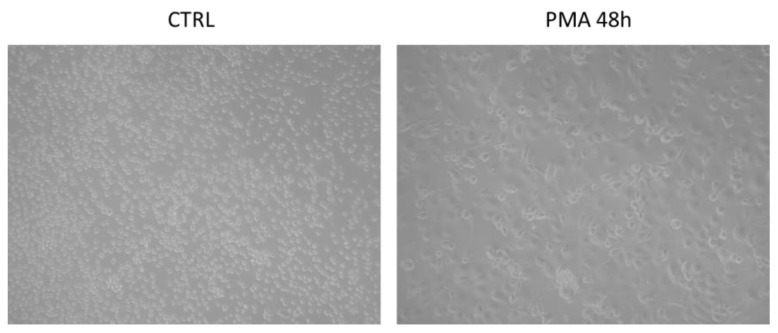
PMA-induced differentiation in THP-1 monocytes. THP-1 cells were incubated for 48 h with or without PMA (100 ng/mL).

To induce differentiation of THP-1 cells into macrophage-like ones, monocytes were placed into six-well plates at a cell density of 2 × 10^5^ cells/mL and treated with 100 ng/mL of phorbol 12-myristate 13-acetate (PMA) for 48 h [58]. After attachment on the bottom surface, PMA-contained medium was removed and THP-1 macrophages were subsequently cultivated for further 24 h in PMA-free culture medium prior starting the experiments. A representative image of THP-1 differentiated macrophages and undifferentiated are shown in Figure 9. Each cell culture reagent was from Gibco (Life Technologies, Monza, Italy). OLC and PMA were from Sigma (Milan, Italy), while LPS was from InvitroGen (San Diego, CA, USA).

In our experiments, THP-1-derived macrophages were seeded at a density of 5 × 10^5^ cells/mL into culture plates in RPMI complete medium plus 10% FBS and incubated at 37 °C with LPS at 100 ng/mL concentration for 24 h, in the presence or absence of OLC, which was added to the culture medium 30 min prior to the LPS treatment. 

### 4.2. Cell Viability Assay

Cell viability was evaluated by the MTT test as reported by Morisi et al. [59]. THP-1 differentiated macrophages were seeded into 96-well plates at density of 5 × 10^4^ cells/well and left to attach overnight. Then, the cells were treated as previously described. Afterwards, the plates were centrifuged at 1200 rpm for 10 min, supernatants were removed and fresh media without phenol red containing 0.5 mg/mL of MTT was added to each well. Plates were put in the incubator for additional 4 h. Then, insoluble formazan crystals were dissolved in 100 mL HCl/isopropanol 0.1 N lysis solution. The absorbance was spectrophotometrically quantified through iMark™ microplate reader (Bio-Rad Laboratories, Milan, Italy) at a wavelength of 570 nm with reference at 630 nm. The viability was determined as the percentage of viable cells in treated cultures compared to the percentage in untreated ones. 

### 4.3. Evaluation of Cytokine Secretion by ELISA Assay

In order to detect human IL-6, IL-1β, TNF-α, IL-10, COX-2 and PGE_2_, an enzyme-linked immunosorbent assay was performed in *cell-free* culture supernatants of THP-1-derived macrophages, using Instant ELISA Kits (eBioscience, Vienna, Austria). Firstly, supernatants from treated and untreated cells were concentrated 10-fold by freeze-drying. All freeze-dried samples were reconstituted by the addition of distilled water. Then, according to the manufacturer’s guidelines, 50 µL of standards or samples were incubated in 96-well plates at room temperature for 3 h with shaking. After washing them five times with 400 µL of wash buffer, 100 µL of the substrate solution were added to each well and the plates were incubated in the dark for 10 min. The enzyme reaction was then stopped by 100 µL of stop solution in each well, and the absorbance was recorded at 450 nm using a microplate reader. All experiments were performed in triplicate. 

### 4.4. Evaluation of NO by Griess Reaction

NO was detected in the cell supernatant according to the method of Green et al. [60]. THP-1-derived macrophages were treated as previously described and then, 100 μL of supernatant were collected, centrifuged at 1000 rpm and incubated with an equal volume of Griess reagent (0.1% N-1-napthylethylenediamine dihydrochloride and 1% sulphanilamide in 5% phosphoric acid) for 10 min at room temperature. Absorbance was measured at 540 nm by a microplate reader. Nitrite concentration was calculated using a standard sodium nitrite curve.

### 4.5. Real-Time PCR Analysis

For the evaluation of IL-6, IL-1β, TNF-α, IL-10, CD14, TLR4 and MyD88 gene expression, THP-1-derived macrophages were treated as previously described. Afterwards, the total RNA of treated or untreated cells was extracted by TRIzol reagent (Invitrogen Life Technologies, Milan, Italy) and 2 µg was reverse transcribed with High-Capacity cDNA Archive kit (Applied Biosystems, Applera Corp., Milan, Italy), according to the manufacturer’s instructions. Messenger RNA levels were analyzed by quantitative real-time PCR, using SYBR green as a fluorescent probe. The analysis was carried out on a 7300 Real-Time PCR System (Applied Biosystems). β-Actin was used as housekeeping control. Data were collected and analyzed using the 2^-ΔΔCT^ relative quantification method [61]. Values were presented as fold change relative to untreated cells. The primer sequences used for the real-time PCR are listed in Table 2.

### 4.6. TLR4/CD14 Co-Staining

The expression of TLR4 and CD14 in THP-1-derived macrophages was determined by flow cytometry. Briefly, after treatment with OLC 1, 2.5 and 5 µM 30 min prior LPS stimulation (100 ng/mL), cells (1 × 10^6^) were washed with PBS, then resuspended in 100 µL of PBS and incubated at 4 °C for 30 min adding 5 µL of both anti-CD14 FITC-conjugated (eBioscience, Life Technologies, Carlsbad, CA, USA) and anti-TLR4 PE-conjugated antibodies (eBioscience). Afterwards, cells were washed and fluorescence of at least 10,000 events analyzed by a Novocyte 2000 cytofluorimeter (Agilent, Santa Clara, CA, USA).

### 4.7. Assessment of Protein Levels of MyD88

After treatments, THP-1-derived macrophages were harvested and lysed by lysis buffer (50 mM Tris-HCl, pH 7.4, 1 mM EDTA, 100 mM NaCl, 20 mM NaF, 3 mM Na_3_VO_4_, 1 mM PMSF and protease inhibitor cocktail). Lysates were then centrifuged at 10,000× *g* for 5 min, and the supernatants were collected and analyzed for quantification of Myd88 through human MyD88 ELISA Kit (ab171341; Abcam, Cambridge, UK). Briefly, an antibody against MyD88 was pre-coated onto a 96-wellplate and blocked. Standards or samples were added to the wells and incubated for 1 h. After washing, a biotinylated detector antibody specific to MyD88 was added and incubated, followed by washing for 30 min. Avidin-HRP conjugate was then added and incubated and unbound conjugate was washed away. An enzymatic reaction was produced through the addition of TMB substrate, which is catalyzed by HRP, generating a blue color product that changes in yellow after adding acidic stop solution. The optical density of yellow coloration (450 nm) was quantitatively proportional to the amount of MyD88 captured in well. Data were expressed as a percentage of the control.

### 4.8. NF-κB p65 Activity Assay

THP-1-derived macrophages were treated as previously described and were then harvested, and their nuclear extracts were prepared using the Nuclear Extraction Kit (ab113474; Abcam, Cambridge, UK) following the manufacturer’s instructions. The activation of NF-κB was assayed using the ELISA-based TransAM NF-κB p65 kit (Active Motif, Carlsbad, CA, USA), following the manufacturer’s protocol. The NF-κB TransAM kit contains a 96-well plate with immobilized oligonucleotides encoding an NF-κB consensus site (5′-GGGACTTTCC-3′). The active form of NF-κB contained in the nuclear cellular extracts specifically binds the oligonucleotide. The primary antibody, used to detect NF-κB, recognizes an epitope on p65 that is accessible only when NF-κB is activated and bound to its target DNA sequence. Briefly, nuclear extracts were then added into black 96-well plates containing the consensus binding sequence for NF-κB p65 and incubated with shaking for 1 h at room temperature. After washing, NF-κB specific primary antibody was loaded into each well and incubated for 1 h at room temperature. Then, after washing, the HRP-conjugated secondary goat anti-rabbit antibody was added into each well and incubated for 1 h at room temperature. Each well was washed five times and loaded with a developing solution and then incubated for 30 min at room temperature. The absorbance was read in a microplate reader (Bio-Rad Laboratories) at 450 nm with a reference wavelength set to 655 nm, following the addition of stop solution to each well. Data were expressed as a percentage of the control.

### 4.9. Evaluation of OLC Anti-Oxidant Activity through Abiotic Assays

The total antioxidant activity of OLC was evaluated by the oxygen radical absorbance capacity (ORAC) assay, following Cirmi et al. [62]. The DPPH assay was employed to test the radical scavenging activity of OLC [63]. The reducing power of OLC was determined according to Lombardo et al. through the PFRAP assay [64]. The results are reported as mean ± SEM of three experiments performed in triplicate and expressed in standard equivalent/g of OLC. As standards, we employed Trolox and ascorbic acid, in relation to the assay performed.

### 4.10. DCFH_2_-DA Staining

Intracellular ROS were quantified employing 2′,7′-dichlorodihydrofluorescein diacetate (DCFH_2_-DA, Sigma-Aldrich) [65]. Firstly, cytosolic esterases hydrolyze the acetate group bonds of DCF-DA releasing the non-fluorescent moiety DCF H_2_, which in turn is oxidized by intracellular ROS to produce a highly fluorescent compound (DCF). THP-1-derived macrophages were treated with OLC 1, 2.5 and 5 µM, 30 min prior to being exposed to LPS (100 ng/mL) for 24 h. Each sample was centrifuged at 1200 rpm, washed with PBS and resuspended in serum-free RPMI 1640 medium with 10 µM DCFH-DA for 30 min at 37 °C. Afterwards, cells were washed and resuspended in PBS, and fluorescence of at least 10,000 events was measured with Novocyte 2000 cytofluorimeter (Agilent) at 495 nm excitation and 520 nm emission.

### 4.11. Statistical Analysis 

One-way analysis of variance (ANOVA) was used to interpret the data. Multiple comparisons of the means of the groups were performed using the Tukey–Kramer test (SigmaPlot Version 12.0, Systat Software, San Jose, CA, USA).

## 5. Conclusions

In the presents study, we firstly demonstrated the anti-inflammatory effects of OLC, which attenuated LPS-induced inflammation and inhibited the activation of TLR4/MyD88/NF-κB signaling in THP-1 macrophages, along with the related downstream mediators. Moreover, we demonstrated OLC anti-oxidant activity both in in vitro and in cell-free models. Overall, our results suggest that the anti-inflammatory and anti-oxidant activities of OLC may cooperate in its protective effect against inflammatory stressors as LPS, and support the hypothesis of OLC employment as a possible alternative pharmacological strategy aimed at reducing the inflammatory process.

## Figures and Tables

**Table 1:** Antioxidant activity of OLC evaluated by abiotic assays. The results are reported as mean ± SEM of three experiments performed in triplicate and expressed in standard equivalent/g of OLC.

DPPH (mg TE/g)	68.37± 1.28
PFRAP (mg AAE/g)	80.14 ± 1.31
ORAC (µmol TE/g)	70.65 ± 1.13

**Table 2:** Oligonucleotide primer sequences used for the real-time PCR.

Gene	NCBI Reference Sequence	Primer Sequence
*IL*-*6*	NM_000600.5	Forward: 5′- CCACCGGGAACGAAAGAGAA -3′ Reverse: 5′- GAGAAGGCAACTGGACCGAA -3′
*IL*-*1β*	NM_000576.3	Forward: 5′- AGCCATGGCAGAAGTACCTG -3′ Reverse: 5′- TGAAGCCCTTGCTGTAGTGG -3′
*TNF*-*α*	NM_000594.4	Forward: 5′- CACAGTGAAGTGCTGGCAAC -3′ Reverse: 5′- ACATTGGGTCCCCCAGGATA -3′
*IL*-*10*	NM_000572.3	Forward: 5′- AGACAGACTTGCAAAAGAAGGC -3′ Reverse: 5′- GGCAACCCAGGTAACCCTTA -3′
*CD14*	NM_001174105.2	Forward: 5′- TTCTGAGGGTCCTCGTCAAC -3′ Reverse: 5′- CGTGTGGATCCTGAGGGTTA -3′
*TLR4*	NM_003266.4	Forward: 5′- TGGGCAACCTGCTCTACCTA -3′ Reverse: 5′- GCTGTAGCT CGTTGGCAGA -3′
*MyD88*	NM_001172567.2	Forward: 5′- GCATATGCCTGAGCGTTTCG -3′ Reverse: 5′- TTCTGATGGGCACCTGGAGA -3
*β-actin*	NM_001101.5	Forward: 5′- TTGTTACAGGAAGTCCCTTGCC -3′ Reverse: 5′- ATGCTATCACCTCCCCTGTGTG -3′

## Data Availability

The datasets generated for this study are available on request to the corresponding author.
